# Comparative study of the radiosensitising and cell cycle effects of vinflunine and vinorelbine, *in vitro*

**DOI:** 10.1186/1471-2407-8-65

**Published:** 2008-02-29

**Authors:** Cindy Simoens, Filip Lardon, Bea Pauwels, Christel MJ De Pooter, Hilde AJ Lambrechts, Greet GO Pattyn, Fabienne Breillout, Jan B Vermorken

**Affiliations:** 1 Laboratory of Cancer Research and Clinical Oncology, Department of Medical Oncology, University of Antwerp (UA/UZA), Wilrijk (Antwerp), Belgium; 2 Department of Radiotherapy, St. Augustinus Hospital, Wilrijk (Antwerp), Belgium; 3 Institut de Recherche Pierre Fabre, Centre de développement Oncologie, Boulogne Cedex, France

## Abstract

**Background:**

Vinca alkaloids are an important class of anticancer agents and semisynthetic vinca alkaloids are developed to improve the therapeutic index of this class of drugs. In the present study, a direct comparison was made between vinflunine and vinorelbine regarding their radiosensitising and cell cycle effects.

**Methods:**

Four human tumour cell lines were tested under identical experimental conditions, using equitoxic concentrations of vinflunine and vinorelbine.

**Results:**

Vinflunine and vinorelbine induced a comparable radiosensitising effect (p-value never below 0.01) when cells were incubated for 24 h immediately prior to radiation. Regarding the cell cycle effects, a statistically significant concentration-dependent G2/M block was seen after 24 h incubation with vinorelbine in all tested cell lines. Similar results, with small cell line-related differences, were observed with vinflunine.

**Conclusion:**

The radiosensitising effects of both semisynthetic vinca alkaloids were comparable (not statistically different) and nearly always cell line-specific and concentration-dependent. The cell cycle effects could be related to the observed radiosensitising effects. Considering the more favourable toxicity profile of vinflunine, this agent might be more promising than vinorelbine for chemoradiation studies in the clinic.

## Background

Vinca alkaloids are an important class of antitumour agents used in chemotherapy, either as single agents or in combination regimens. Semisynthetic vinca alkaloids, such as vinorelbine (VRL) and most recently vinflunine (VFL), have been developed to improve the therapeutic index [1]. Like the other vinca alkaloids, VRL and VFL exert their antitumour effect by binding to tubulin, the major component of microtubules in mitotic spindles. They diminish microtubule dynamics and assembly, which ultimately results in cell cycle arrest at the metaphase/anaphase transition [2,3]. However, they also differ from the naturally occurring vinca alkaloids in chemical structure, microtubule selectivity, and toxicity. In that respect, the capacity of VRL to bind preferentially to mitotic rather than axonal microtubules may imply that occurrence of neurotoxicity is less likely with VRL than with vinblastine or vincristine, and may predict a wider therapeutic window for VRL [4]. VFL also displays some differences in the interaction with microtubules, compared to all the other vinca alkaloids, which may lead to different effects on cell cycle progression and cell death [5-7]. In the clinic, both VRL and VFL have demonstrated activity i.e. in the treatment of non-small cell lung (NSCLC), metastatic breast (MBC) and bladder cancer [8-13].

### Vinflunine versus vinorelbine

Although VFL has a structure close to that of VRL, the selective introduction of the two fluorine atoms seemed to influence the drugs characteristics:

- VFL's potency with respect to both inhibition of cell proliferation and mitotic block is lower than that of VRL [6]. VFL exhibits a relatively low *in vitro *cytotoxic potency, but has shown superior *in vivo *activity against a series of murine and human tumour experimental models [14-16]. These data may suggest a wider spectrum of activity for VFL and studies are underway to evaluate if this enhanced efficacy can be translated into an improved spectrum of clinical activity for VFL [16].

- VFL also has a 3–16 fold lower overall binding affinity for tubulin than VRL [17]. The lower affinity of VFL may explain, at least in part, the high concentrations of the drug needed to block mitosis and cell proliferation [18]. These findings do not explain the relatively high therapeutic efficacy of VFL. However, the therapeutic index of a drug with a relatively weak potency may be broader than that of an extremely potent drug [6]. In this context, the NMR study of Fabre et al. [19] revealed the presence of specific binding sites and showed a different affinity of VFL and VRL to the tubulin dimer at physiological temperatures. This could account for their different toxicity, and not necessary implies a different mechanism of action between these compounds, and is in agreement with in vivo and in vitro observations.

- The peak intracellular drug concentrations at the mitotic IC50-value are highest for VFL (4.2 ± 0.2 μM vs 1.3 ± 0.1 μM for VRL). This suggests that intracellular binding reservoir(s) may be partially responsible for VFL's high efficacy, by providing a reservoir for excess drug and enabling its gradual release [6]. VFL also induces significantly smaller spirals (tubulin aggregates) than VRL and demonstrates shorter relaxation times compared with VRL [17]. This lower overall binding affinity for tubulin, together with the smaller spirals, the shorter relaxation times and the gradual release, suggest that VFL may demonstrate reduced neurotoxicity relative to VRL [17].

- Finally, VFL seems to be a far less potent inducer of drug resistance relative to VRL [15,20].

### Chemoradiation

There is increasing interest in the combined use of chemotherapy and radiotherapy in the clinic for various tumour types, such as NSCLC, head and neck, oesophageal and cervical cancer [21-25]. Improved outcomes in patients are most likely a result of increased systemic and local tumour control, and from a direct interaction between cytotoxic agents and radiation. This latter aspect should preferably be investigated *in vitro *first in order to optimise the clinical application of the combination.

The *in vitro *ability of VRL to potentiate radiation was already evaluated in several human NSCLC cell lines (NCI-H460, A549 & PC9) [26,27], SCLC cells (SBC-3) [28], and head & neck carcinoma cell lines [29]. The results were cell line-dependent, and showed an additive effect or a dose-dependent potentiation of radiation. In a previous study, we investigated the *in vitro *ability of VFL to potentiate radiation in 4 different human tumour cell lines (bladder, head & neck, breast and lung tumour cell lines). A dose-dependent radiosensitising effect was shown after 24 h treatment with VFL immediately prior to radiation, in all cell lines [30].

In the current study, the radiosensitising and cell cycle effects of VFL and VRL are compared, using the same set of 4 human tumour cell lines and identical experimental conditions for both drugs. In this manner, the radiosensitising effect of vinflunine can be placed in a more identifiable context.

## Methods

### Cell lines

Four different human tumour cell lines were used in this study: ECV304, an epidermoid bladder cancer cell line; CAL-27, a squamous cell carcinoma cell line of the tongue; MCF-7, a breast cancer cell line; and H292, a mucoepidermoid NSCLC cell line. ECV304 cells were cultured in Medium 199 (Invitrogen, Merelbeke, Belgium); CAL-27 and MCF-7 cells in DMEM medium (Invitrogen), supplemented with 2 mM glutamine (Invitrogen); and H292 cells in RPMI-1640 medium (Invitrogen), supplemented with 2 mM glutamine and 1 mM sodium pyruvate (Invitrogen). All media were completed with 10% foetal calf serum (Invitrogen), no antibiotics were added. Cultures were maintained in exponential growth at 37°C in a humidified 5% CO_2 _atmosphere.

### Vinorelbine and vinflunine

VRL and VFL were kindly provided by the 'Institut de Recherche Pierre Fabre', Boulogne, France. For VRL, each vial consisted of 1 ml containing 10 mg free base in solution (i.e. 10 mg/ml); for VFL, each vial consisted of 2 ml containing 50 mg free base in solution (i.e. 25 mg/ml). Both VRL and VFL were diluted in sterile normal saline (0.9% NaCl) to make a stock solution of 3 μM and 30 μM respectively, and were stored at 4°C (no longer than 2 months). Before use, the stock solutions were further diluted in 0.9% NaCl to the desired concentration.

### Cytotoxicity and chemoradiation experiments

Cells were harvested from exponential phase cultures (at 50–75% confluence) by trypsinisation, counted and plated at optimal seeding densities in 48-well plates to assure exponential growth during the experiments. Seeding densities were about 1200, 2000, 2200 & 2800 cells/well in the cytotoxicity experiments and about 250, 100, 150 & 120 cells/well in the chemoradiation experiments, for ECV304, CAL-27, H292, and MCF-7, respectively. After a 24 h recovery period, cells were treated during 24 h with VRL or VFL. For determining the cytotoxic effect, a concentration range of both compounds was tested (0–80 nM VRL/0–400 nM VFL, dissolved in 0.9% NaCl). After the incubation period, cells were washed with drug free medium and cell survival was determined by the sulforhodamine B (SRB) assay 4 days after the start of treatment. In the chemoradiation experiments, cells were treated with specified concentrations of VRL or VFL (see Table 1) and this was immediately followed by radiation (Cobalt-60 γ rays, 0–8 Gy, room temperature). Experiments were started with low VFL concentrations and these were increased until a clear radiosensitising effect was observed. For VRL, about equitoxic concentrations were applied. After radiation, cells were washed with drug free medium and incubated at 37°C for 7 or 8 days (about 6 doubling times). Cell survival was determined again by the SRB assay, a reliable assay in these circumstances, as described previously [31].

0.9% NaCl alone was added to control cells. Each concentration was tested 6 times within the same experiment and all experiments were performed at least 3 times. The SRB assay was performed according to the method of Skehan et al. and Papazisis et al. [32,33], with minor modifications [30].

### Cell cycle experiments

Exponential growing cells (at 50–75% confluence) were trypsinised, counted and plated in 6-well plates. In order to assure exponential growth during the experiments, seeding densities were about 75 000 cells/well for the first set of experiments, and about 50 000 cells/well for the cell cycle kinetics experiments. After at least a 24 h recovery period, two different cell cycle experiments were performed as follows:

- Firstly, the effect of VRL or VFL on the cell cycle was investigated. For these experiments, cells were incubated for 24 h with specified concentrations of VRL or VFL (0–50 nM/0–400 nM) and flow cytometry was performed immediately after incubation.

- Secondly, the cell cycle was investigated over time (cell cycle kinetics). For these experiments, not only different incubation times were investigated (4 – 48 h), but also different time points after a 24 h incubation (3 – 72 h). Therefore cells were treated during 24 h with VRL or VFL and cell cycle analysis was performed 0, 3, 24, 48 or 72 h after drug wash out (indicated as 24+0, 24+3, 24+24, 24+48, 24+72, respectively). The concentrations used for this second set of experiments were identical to those resulting in a clear G2/M block in the first set of experiments with VFL, and their equitoxic VRL concentrations, i.e. 30 nM VRL/150 nM VFL for ECV304 and H292, 15 nM VRL/150 nM VFL for MCF-7 and 15 nM VRL/100 nM VFL for CAL-27 cells.

Cell cycle analysis was performed by flow cytometry, after DNA staining, according to the Vindelov method [34], as described previously [30].

### Data analysis and statistics

#### Cytotoxicity and chemoradiation experiments

The survival rates were calculated by: mean optical density (OD) of treated cells/mean OD of untreated cells × 100%. The survival curves after treatment with VRL or VFL alone were fitted according to the sigmoid inhibition model: exp(survival) = 1-(C^γ^/C^γ^+IC50^γ^). The radiation dose-survival curves were fitted according to the linear-quadratic model: survival = exp(-αD - βD^2^), using WinNonlin (Pharsight, Palo Alto, CA, USA). The radiation dose-survival curves were corrected for the cytotoxic effect of VRL or VFL alone. From these dose-survival curves, the following parameters were calculated: the IC50, i.e. the concentration VRL or VFL causing 50% growth inhibition; the ID50, i.e. the radiation dose causing 50% growth inhibition; and the mean inactivation dose (MID), which was calculated by numerical integration of the linear-quadratic curve [35]. A two-sample t-test was used to investigate significant differences at the level of ID50 and MID values, between control cells (only irradiated) and cells treated with the combination of chemo- and radiotherapy. The same statistical analysis was also performed to compare equitoxic concentrations of VRL with VFL in the chemoradiation experiments. Statistical significance was defined at the level of p < 0.01. The results are expressed as mean ± standard error. Radiosensitisation was represented by the dose enhancement factor (DEF): ID50 (without VRL or VFL)/ID50 (with VRL or VFL). Possible synergism was determined by calculation of the combination index (CI) by the Chou and Talalay equation [36], using CalcuSyn (Biosoft, Cambridge, UK), which can be used for chemoradiation combinations [37]. The CI quantifies drug interaction in terms of additive effect (CI = 1), synergism (CI < 1), or antagonism (CI > 1). The CI takes into account both the potency (IC50 or D_m_) and the shape of the dose-survival curve (m value, signifying the sigmoidicity of the dose-effect curve). The general equation for the classic isobologram is given by:

CI = (D)_1_/(D_x_)_1 _+ (D)_2_/(D_x_)_2_

where (D_x_)_1 _and (D_x_)_2 _are the doses (or concentrations) for D_1 _(VRL or VFL) and D_2 _(radiation) alone, required to inhibit cell growth by 50%, and (D)_1 _and (D)_2 _are the doses of VRL or VFL and radiation in combination that also inhibit cell growth by 50% (i.e., isoeffective as compared with the single treatments).

The (D_x_)_1 _or (D_x_)_2 _(for VRL or VFL and radiation) are calculated by the formula:

D_x _= D_m _[f_a_/(1-f_a_)]^1/m^

Where D_m _is the dose required to produce absorbance readings 50% lower than those of non-treated wells (IC50 or ID50), f_a _is the fraction affected and m is the slope of the median-effect plot. The CI values obtained from the classic (mutually exclusive) isobologram calculations were used. In short: 1.10 > CI > 0.90, 0.90 > CI > 0.85, 0.85 > CI > 0.70 and 0.70 > CI > 0.30 indicating additivity, slight synergism, moderate synergism and synergism, respectively.

#### Cell cycle experiments

Flow cytometric data were analysed using Cell Quest (Becton Dickinson). In our experiments, polyploid cell populations appeared after treatment with both VRL and VFL. Therefore, besides the normal cell cycle phases G1, S and G2/M; S_2 _(second synthesis phase, without previous mitosis) and polyploid G2/M ((G2/M)_2_, cells in G2/M after S_2_), with a double DNA content compared to cells in normal G2/M, were explored.

A 2 sample t-test was used to investigate the significance of the differences between the percentages of cells in the different cell cycle phases after treatment with VRL or VFL versus the untreated cells, 24 h incubation, 24+0 h, and 24+24 h schedule (see previous section for the explanation of the different treatment schedules), and to compare equitoxic concentrations of VRL with VFL. Statistical significance was defined at the level of p < 0.05. The results are expressed as mean ± standard error.

## Results

### Cytotoxicity experiments

To investigate and compare the cytotoxic effect of VRL and VFL, IC50-values were calculated for both drugs. All tested cell lines were 5–8 times more sensitive to VRL than to VFL (data not shown). These results enabled us to use equitoxic concentrations of VRL and VFL to directly compare the radiosensitising and cell cycle effects of these two compounds.

### Chemoradiation experiments

The radiation parameters of the 4 human tumour cell lines treated with radiation alone or with the combination of VRL and radiation are summarised in Table 1. The combination of VRL and radiation resulted in more cell kill compared to the control curves (irradiation only) in all 4 cell lines. In ECV304, radiosensitisation was already seen at concentrations around IC15 (DEF = 1.49), resulting in slight synergism using CI calculations. For H292, concentrations around IC20 were required (DEF = 1.36) to observe a moderate synergistic effect, and for CAL-27 cells, concentrations around IC45 (DEF = 1.92) resulted in clear synergism. With higher VRL concentrations, an increase in radiosensitivity was observed in all these cell lines (higher DEFs, accompanied by a decrease of ID50 and MID values). In MCF-7 however, once a radiosensitising effect was established (from low concentrations on), the effect was not dose proportional with VRL. The resulting DEF (± 1.5) was equal for every tested concentration (between IC20 and IC55), although CI calculation showed an increase from an additive effect to moderate synergism.

**Table 1:** Radiation parameters of the four human tumour cell lines, for VRL and VFL; mean values ± standard error.

Cell line	Conc. (nM)	IC-value*	N	Mean MID	Mean ID50	Mean DEF	Mean CI	
	**VRL**							
ECV304	0		13	3,23 ± 0,14	2.71 ± 0.15			
	4	IC15	8	2,45 ± 0,24^a^	1.88 ± 0.23^a^	1.49 ± 0.13	0.89 ± 0.01	*slight synergism*
	6	IC30	9	2,10 ± 0,21^a^	1.56 ± 0.16^a^	1,97 ± 0,26	0,77 ± 0,01	*moderate synergism*
CAL-27	0		9	3,23 ± 0,12	2.99 ± 0.12			
	2,5	IC45	9	2,22 ± 0,20^a^	1.68 ± 0.19^a^	1,92 ± 0,24	0,69 ± 0,01	*synergism*
	3,5	IC75	8	1,81 ± 0,24^a^	1.33 ± 0.17^a^	2,49 ± 0,41	0,51 ± 0,01	*synergism*
MCF-7	0		19	3.26 ± 0.12	2.80 ± 0.16			
	3	IC20	17	2.66 ± 0.17^a^	2.00 ± 0.15^a^	1.48 ± 0.05	0.93 ± 0,02	*additivity*
	4	IC55	11	2.55 ± 0.28^a^	1.94 ± 0.21^a^	1.47 ± 0.10	0.78 ± 0,04	*moderate synergism*
H292	0		16	3,69 ± 0,10	2.91 ± 0.10			
	3	IC10	15	3,18 ± 0,18	2.51 ± 0.16	1,19 ± 0,06	0,97 ± 0,02	*additivity*
	4	IC20	5	2,59 ± 0,27^a^	2.00 ± 0.22^a^	1,36 ± 0,06	0,83 ± 0,04	*moderate synergism*
								
	**VFL**							
ECV304	0		5	3.18 ± 0.25	2.46 ± 0.28			
	30	IC10	5	2.12 ± 0.26	1.56 ± 0.16	1.57 ± 0.11	0.84 ± 0.01	*moderate synergism*
	50	IC25	5	1.66 ± 0.08^a^	1.26 ± 0.05^a^	1.93 ± 0.14	0.71 ± 0.01	*moderate synergism*
CAL-27	0		5	3.24 ± 0.13	2.81 ± 0.17			
	25	IC40	4	2.48 ± 0.23	2.01 ± 0.26	1.41 ± 0.09	0.98 ± 0.04	*additivity*
	30	IC70	4	1.63 ± 0.06^a^	1.22 ± 0.05^a^	2.29 ± 0.23	0.57 ± 0.02	*synergism*
MCF-7	0		4	4.00 ± 0.34	3.18 ± 0.26			
	30	IC40	4	3.07 ± 0.38	2.29 ± 0.29	1.42 ± 0.13	0.77 ± 0.01	*moderate synergism*
	40	IC60	4	1.93 ± 0.16^a^	1.48 ± 0.14^a^	2.24 ± 0.08	0.54 ± 0.01	*synergism*
H292	0		4	5.16 ± 0.36	4.42 ± 0.31			
	30	IC10	4	4.55 ± 0.64	3.55 ± 0.46	1.29 ± 0.14	1.27 ± 0.12	*additivity*
	40	IC40	3	3.60 ± 0.21^a^	3.03 ± 0.20^a^	1.53 ± 0.02	0.77 ± 0.03	*moderate synergism*

N = number of experiments

MID = mean inactivation dose

ID50 = radiation dose causing 50% growth inhibition

DEF = Dose Enhancement Factor

CI = Combination Index

^a ^p < 0.01 compared to control (0 nM VFL)

* IC-value represents the concentration of VRL or VFL causing a specific percentage of inhibition of cell growth

The radiation dose-survival curves of VFL have been presented earlier and indicated a radiosensitising effect in all tested cell lines [30]. The MID, ID50, DEF and CI-values for VFL are also summarised in Table 1. The radiosensitising effect of VFL was most pronounced in ECV304 cells, already at concentrations around IC10 (moderate synergism). In MCF-7 and H292, radiosensitisation was seen at concentrations around the IC40 (synergism and moderate synergism, respectively), while rather toxic concentrations around IC70 were required in CAL-27 cells to induce a synergistic effect.

To compare the effects of both VFL and VRL on radiation, equitoxic concentrations of VRL were used. The data suggest that the radiosensitising effects of VFL and VRL are comparable, since statistical analysis – performed to compare equitoxic VRL concentrations with VFL – never reached statistical significant differences (p-value never below 0.01). However, cell line specific and concentration-dependent differences were noticed. A marked difference between the two drugs was observed in the MCF-7 cell line, where, contrary to VFL, the radiosensitising effect of VRL was found to be not concentration-dependent. However, this did not reach statistical significance (borderline) since the p-value was only 0.08 comparing 4 nM VRL with 40 nM VFL.

### Cell cycle experiments

**Figure 1:**
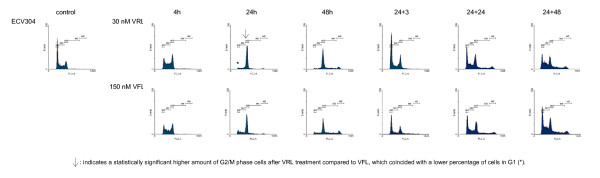
**DNA histograms of vinorelbine and vinflunine in ECV304 cells**. *detailed legend*: DNA histograms of ECV304 cells treated with equitoxic concentrations of vinorelbine or vinflunine during 4, 24 and 48 hours and 24 and 48 h after drug removal. FL-2A = DNA content. Events = number of fluorescent nuclei.

**Figure 2:**
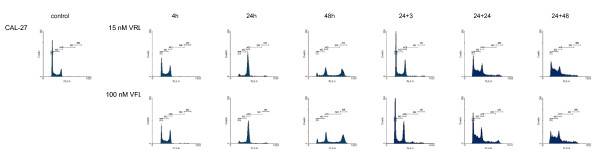
**DNA histograms of vinorelbine and vinflunine in CAL-27 cells**. *detailed legend*: DNA histograms of CAL-27 cells treated with equitoxic concentrations of vinorelbine or vinflunine during 4, 24 and 48 hours and 24 and 48 h after drug removal. FL-2A = DNA content. Events = number of fluorescent nuclei.

**Figure 3:**
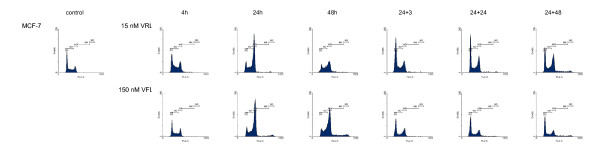
**DNA histograms of vinorelbine and vinflunine in MCF-7 cells**. *detailed legend*: DNA histograms of MCF-7 cells treated with equitoxic concentrations of vinorelbine or vinflunine during 4, 24 and 48 hours and 24 and 48 h after drug removal. FL-2A = DNA content. Events = number of fluorescent nuclei.

**Figure 4:**
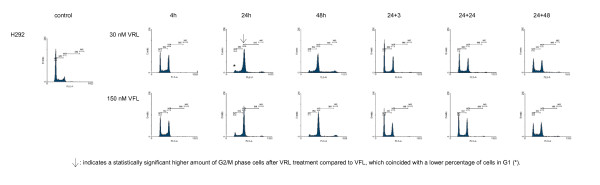
**DNA histograms of vinorelbine and vinflunine in H292 cells**. *detailed legend*: DNA histograms of H292 cells treated with equitoxic concentrations of vinorelbine or vinflunine during 4, 24 and 48 hours and 24 and 48 h after drug removal. FL-2A = DNA content. Events = number of fluorescent nuclei.

Figures 1, 2, 3, 4 show representative DNA-histograms of all tested cell lines after different time points of exposure to VRL or VFL, and at different time points after drug removal.

#### Cell cycle effects after 24 h treatment with vinorelbine or vinflunine

Table 2 summarises the percentages of cells in the different phases of the cell cycle (G1, S & G2/M) for all tested cell lines, treated with a low and a high VRL concentration, which approximates an equitoxic VFL concentration of 60 and 150 nM (these VFL concentrations were selected because they showed a significant G2/M block in these cell lines in previous experiments). Immediately after 24 h treatment with VRL, a concentration-dependent G2/M block was observed in all cell lines (Figures 1, 2, 3, 4, Table 2). In ECV304, a statistically significant G2/M block was observed with 30 nM VRL (high VRL concentration), which coincided with a significant decrease of cells in G1 and S phase. No clear cell cycle effect was observed with lower concentrations. In CAL-27, MCF-7 and H292, a statistically significant G2/M block was apparent with the low VRL concentrations, S phase cells were unchanged, but cells in G1 were decreased. The amount of cells arrested in G2/M increased with increasing concentration, although this effect was only moderately expressed in MCF-7 cells. As in ECV304, these higher VRL concentrations caused statistically significant changes in the amount of G1 and S phase cells. Similar results were observed with VFL [30].

**Table 2:** Cell cycle distributions after 24 h incubation with equitoxic concentrations of VRL and VFL.

Cell line	Contr	60 nM VFL	Equitoxic conc. VRL	150 nM VFL	Equitoxic conc. VRL
	% G1				
ECV304	39.1 ± 1.9	36.3 ± 2.2	*(10 nM) *38.6 ± 2.8	15.0 ± 3.4^a^	*(30 nM) *10.1 ± 1.6^a^
CAL-27	56.4 ± 1.7	19.0 ± 1.0^a^	*(10 nM) *19.0 ± 3.4^a^	9.2 ± 1.1^a^	*(30 nM) *8.1 ± 1.5^a^
MCF-7	50.3 ± 1.5	19.1 ± 2.3^a^	*(8 nM) *18.0 ± 1.7^a^	11.3 ± 3.1^a^	*(20 nM) *10.9 ± 1.8^a^
H292	56.5 ± 0.9	39.3 ± 2.4^a^	*(15 nM) *25.6 ± 3.0^a ^*	15.0 ± 3.0^a^	*(40 nM) *9.3 ± 2.6^a^
					
	% S				
ECV304	38.6 ± 1.6	44.1 ± 2.9	*(10 nM) *37.1 ± 1.6	26.4 ± 3.2^a^	*(30 nM) *17.4 ± 1.7^a^
CAL-27	25.0 ± 1.1	33.3 ± 2.4	*(10 nM) *31.0 ± 3.1	14.9 ± 1.9^a^	*(30 nM) *18.8 ± 2.6
MCF-7	26.2 ± 1.1	26.4 ± 1.4	*(8 nM) *32.1 ± 2.0	29.7 ± 2.2	*(20 nM) *32.1 ± 2.1^a^
H292	23.4 ± 0.5	23.5 ± 1.0	*(15 nM) *20.0 ± 2.8	23.8 ± 2.3	*(40 nM) *18.2 ± 1.8^a^
					
	% G2/M				
ECV304	20.2 ± 1.0	19.2 ± 1.0	*(10 nM) *19.6 ± 2.8	50.2 ± 1.7^a^	*(30 nM) *62.5 ± 2.9^a ^*
CAL-27	16.1 ± 0.7	30.4 ± 3.9^a^	*(10 nM) *32.8 ± 5.0^a^	55.4 ± 3.3^a^	*(30 nM) *53.7 ± 2.4^a^
MCF-7	20.3 ± 0.8	47.2 ± 6.1^a^	*(8 nM) *43.9 ± 1.6^a^	52.5 ± 4.7^a^	*(20 nM) *52.2 ± 1.2^a^
H292	17.8 ± 1.0	28.6 ± 1.3^a^	*(15 nM) *50.2 ± 5.0^a ^*	53.2 ± 6.8^a^	*(40 nM) *65.9 ± 5.0^a^

Percentages of cells in the G1, S and G2/M phase of the cell cycle: after 24 h incubation with equitoxic concentrations of VRL and VFL; mean ± standard error.

^a ^p < 0.05 compared to control

* p < 0.05 compared to the equitoxic concentration of VFL

By comparing the results of VRL with those of VFL, a statistically significant higher amount of G2/M phase cells could be observed in ECV304 cells with 30 nM VRL compared to that observed with the equitoxic 150 nM VFL dose. The same trend was observed for both concentrations of VRL in H292 cells. Treatment with 15 and 40 nM VRL resulted in a higher amount of arrested cells in the G2/M phase than with VFL, which coincides with a lower percentage of cells in G1 after VRL treatment compared to VFL. In CAL-27 and MCF-7 cells, no statistical differences were observed for the cell cycle effects of VRL versus VFL after 24 h treatment.

#### Cell cycle kinetics

Table 3 summarises the percentages of cells in the G2/M phase, after treatment with different incubation times with VRL or VFL (4 – 48 h of continuous incubation). In all cell lines, a statistically significant increase in the percentage of cells in G2/M was already visible after 4 h incubation with VRL. A maximal G2/M block was reached after about 16 h incubation in all the tested cell lines. After this maximum was reached, the amount of arrested G2/M phase cells remained unchanged, or slightly decreased towards the 48 h incubation period. In CAL-27, however, a statistically significant decrease of G2/M blocked cells was observed with the 48 h incubation, accompanied by a strong statistically significant increase of cells in the polyploid population at that time (see below).

**Table 3:** Percentages of cells in the G2/M phase of the cell cycle at different incubation times.

	% of cells in G2/M						
Cell line	Control	4 h incub.	6 h incub.	16 h incub.	20 h incub.	24 h incub.	48 h incub.
	**VRL**						
ECV304	22.5 ± 1.3	37.7 ± 1.7^a^	45.4 ± 2.0^a^	58.2 ± 4.3^a^	53.9 ± 6.9^a^	58.7 ± 6.5^a^	46.4 ± 6.2
CAL-27	17.9 ± 1.0	33.3 ± 1.0^a^	40.3 ± 1.6^a^	66.9 ± 5.6^a^	61.9 ± 3.9^a^	64.3 ± 4.2^a^	23.7 ± 3.0^b^
MCF-7	18.1 ± 1.2	23.2 ± 1.0^a^	27.8 ± 1.5^a^	46.3 ± 3.6^a ^*	39.6 ± 2.0^a ^*	42.6 ± 2.6^a^	36.6 ± 2.3*
H292	18.2 ± 1.2	31.6 ± 3.6^a^	46.8 ± 5.9^a^	71.4 ± 1.7^a^	64.8 ± 4.4^a^	64.7 ± 7.5^a^	60.4 ± 3.1
							
	**VFL**						
ECV304	20.6 ± 1.4	32.7 ± 3.3^a^	37.9 ± 3.1^a^	64.1 ± 5.6^a^	44.9 ± 7.3^a^	48.7 ± 6.5^a^	42.4 ± 3.5
CAL-27	17.1 ± 1.5	33.7 ± 2.4^a^	40.9 ± 1.3^a^	61.2 ± 1.2^a^	63.0 ± 6.5^a^	62.7 ± 5.3^a^	21.3 ± 2.9^b^
MCF-7	18.7 ± 0.9	25.5 ± 1.8^a^	30.8 ± 2.2^a^	59.7 ± 1.0^a^	54.6 ± 3.2^a^	48.7 ± 5.9^a^	49.3 ± 2.9
H292	18.7 ± 1.1	37.8 ± 3.2^a^	38.5 ± 4.1^a^	65.4 ± 5.1^a^	66.0 ± 4.5^a^	69.4 ± 3.0^a^	65.7 ± 1.8

Percentages of cells in the G2/M phase of the cell cycle: at different incubation times with VRL and VFL; mean ± standard error.

^a ^p < 0.05 compared to control

^b ^p < 0.05 compared to 24 h incubation

* p < 0.05 compared to the equitoxic VFL concentration

Table 4 summarises the percentages of cells in the G2/M phase of the cell cycle, different hours after drug removal. 3 h after removal of VRL, the percentage of cells in G2/M decreased significantly in all cell lines (p < 0.05 compared to 24+0). This release of cells from G2/M coincided with an increase in the number of cells in G1 (data not shown) and the polyploid cell cycle (Table 5), and suggested that the accumulated cells in G2/M had re-entered the cell cycle with or without mitosis (normal or polyploid cell cycle). 24 h after VRL removal, a stable cell cycle was established again (no significant differences anymore between 24+24 and 24+72) in CAL-27 and MCF-7 cells. In ECV304, the cell cycle distribution had stabilised 48 h after drug removal and the amount of G2/M cells reached control levels again. In H292, it took 72 h or longer to regain a stable cell cycle distribution.

**Table 4:** Percentages of cells in the G2/M phase of the cell cycle at different time points.

	% of cells in G2/M					
Cell line	Control	24+0 h	24+3 h	24+24 h	24+48 h	24+72 h
	**VRL**					
ECV304	18.1 ± 0.9	70.3 ± 2.1^a^	56.0 ± 5.8^b ^*	32.6 ± 1.6^b^	25.1 ± 0.8^c^	21.4 ± 1.5^c^
CAL-27	14.4 ± 1.2	56.0 ± 1.1^a^	30.7 ± 3.3^b^	23.2 ± 1.3^b^	21.5 ± 0.6	24.3 ± 2.3
MCF-7	17.8 ± 1.2	53.5 ± 0.9^a^	28.1 ± 4.4^b^	35.1 ± 3.5^b^	33.1 ± 0.3	28.6 ± 3.2
H292	13.3 ± 0.8	64.0 ± 4.2^a^	41.9 ± 1.2^b^	40.1 ± 1.5^b^	37.1 ± 1.4	27.1 ± 1.5^c^
						
	**VFL**					
ECV304	17.6 ± 0.9	47.3 ± 6.9^a^	28.2 ± 2.3^b^	31.8 ± 1.7^b^	27.8 ± 1.1	23.4 ± 1.7^c^
CAL-27	13.5 ± 0.5	55.7 ± 6.6^a^	31.8 ± 3.6^b^	24.5 ± 2.0^b^	25.0 ± 0.7	27.6 ± 1.0
MCF-7	17.2 ± 1.0	53.3 ± 5.8^a^	36.2 ± 3.8	27.8 ± 3.4^b^	27.3 ± 3.1	29.8 ± 3.3
H292	11.9 ± 1.4	65.7 ± 1.1^a^	39.5 ± 1.0^b^	41.3 ± 2.3^b^	41.0 ± 2.5	26.9 ± 3.9^c^

Percentages of cells in the G2/M phase of the cell cycle: at different time points after a 24 h incubation with VRL or VFL; mean ± standard error.

^a ^p < 0.05 compared to control

^b ^p < 0.05 compared to 24+0 h

^c ^p < 0.05 compared to 24+24 h

* p < 0.05 compared to equitoxic VFL conc.

**Table 5:** Percentages of cells in the polyploid cell cycle.

Cell line	Control	24 h	48 h	24+24	24+48
	Percentage of cells in S_2_				
	**VRL**				
ECV304	1.5 ± 0.2	5.5 ± 0.9^a^	18.5 ± 1.2^a ^*	17.6 ± 2.3^b^	20.1 ± 0.9^b^
CAL-27	1.3 ± 0.1	5.6 ± 1.6	22.9 ± 1.5^a^	12.3 ± 1.4^b^	20.2 ± 0.9^b^
MCF-7	2.2 ± 0.5	2.9 ± 0.8	9.7 ± 3.9	4.4 ± 0.6	8.6 ± 0.5^b^
H292	1.4 ± 0.4	3.0 ± 1.0	7.9 ± 1.4^a^	3.4 ± 0.7	5.7 ± 0.9
	**VFL**				
ECV304	1.3 ± 0.2	7.6 ± 1.3^a^	22.4 ± 0.8^a^	12.6 ± 1.1^b^	16.2 ± 0.6^b^
CAL-27	1.7 ± 0.5	4.3 ± 1.0	21.8 ± 1.4^a^	11.8 ± 0.7	21.8 ± 0.8^b^
MCF-7	2.0 ± 0.3	2.8 ± 1.0	8.8 ± 1.6^a^	5.2 ± 0.4	10.6 ± 1.1^b^
H292	1.0 ± 0.2	2.9 ± 0.5^a^	7.1 ± 0.9^a^	3.6 ± 1.2	4.5 ± 0.5
					
	Percentage of cells in (G2/M)_2_				
	**VRL**				
ECV304	0.3 ± 0.0	1.2 ± 0.2^a^	12.2 ± 1.5^a^	9.8 ± 1.6^b^	10.2 ± 1.3^b^
CAL-27	0.4 ± 0.1	2.2 ± 0.6	37.4 ± 4.8^a^	4.8 ± 0.5^b^	6.1 ± 0.9^b^
MCF-7	0.5 ± 0.1	1.3 ± 0.3	2.1 ± 0.5	2.1 ± 0.7	3.4 ± 0.2^b^
H292	0.3 ± 0.1	1.1 ± 0.3	6.5 ± 1.0^a^	1.4 ± 0.2	2.2 ± 0.2^b^
	**VFL**				
ECV304	0.2 ± 0.0	0.8 ± 0.3	15.6 ± 0.8^a^	8.5 ± 1.3^b^	9.2 ± 1.1^b^
CAL-27	0.3 ± 0.1	2.1 ± 0.5	41.8 ± 4.9^a^	5.4 ± 0.4	7.5 ± 0.5^b^
MCF-7	0.5 ± 0.1	1.3 ± 0.2^a^	4.4 ± 0.8^a^	2.0 ± 0.5	4.4 ± 0.4^b^
H292	0.2 ± 0.0	1.2 ± 0.2^a^	7.0 ± 0.9^a^	1.9 ± 0.4	2.4 ± 0.3^b^

Percentages of cells in the polyploid cell cycle: at different incubation times with VRL or VFL and at different times after drug removal; mean ± standard error.

^a ^p < 0.05 compared to control

^b ^p < 0.05 compared to 24 h incubation

* p < 0.05 compared to equitoxic VFL conc.

Similar cell cycle effects were observed using the same treatment schedules with VFL [30]. Only in ECV304, the G2/M block after 24 h VRL incubation was more pronounced (as shown above), since the maximal G2/M arrest was maintained longer with this agent. Three hours after removal of VRL, the amount of cells in G2/M was therefore still significantly higher in this cell line compared to VFL. However, in MCF-7 cells, the amount of cells blocked in G2/M was statistically lower after VRL treatment (except for the 24 h timepoint) then after VFL treatment.

#### Polyploid cell cycle

Table 5 summarises the percentages of cells in the polyploid cell cycle (S_2 _and (G2/M)_2_) after 24 h and 48 h of incubation with VRL or VFL, and after drug removal (24+24 & 24+48). The results obtained with VRL were similar to those obtained with VFL. After 24 h and even more pronounced after 48 h of continuous incubation, a polyploid cell population was clearly observed in CAL-27 and ECV304 (Figure 1 and 2), and to a lesser degree also in H292 and MCF-7 (p < 0.05 compared to control). Only in ECV304 cells, the amount of S_2 _phase cells was significantly lower after 48 h incubation with VRL compared to VFL.

Even when VRL or VFL was removed after 24 h incubation, not all cells could proceed in a normal cell cycle. 24 h after the VRL or VFL incubation, a polyploid cell cycle was initiated. This was even more pronounced after 48 h in CAL-27 because of the longer doubling time of this cell line.

## Discussion

The strength of this study, thoroughly investigating the radiosensitising and cell cycle effects of both VFL and VRL, is the direct comparison that could be made between the two semisynthetic vinca alkaloids. It is always difficult to compare new results with formerly published results of other research groups, because of the use of other techniques, different experimental set-up, different cell lines, concentrations, etc. Therefore, we used for both VFL and VRL identical experimental conditions and equitoxic concentrations, and performed the experiments in the same four human tumour cell lines. In this manner, the radiosensitising effect of VFL could be placed in a somewhat more identifiable context.

The radiosensitising effect of both semisynthetic vinca alkaloids was dependent on the cell line tested and most of the time also on the concentration used. When comparing the VFL results to those of VRL, only small, statistically insignificant differences were observed. In ECV304 cells, VFL was slightly more radiosensitising than VRL. With a VFL concentration around IC10 the calculated DEF was 1.57 (moderate synergism), while the DEF was 1.49 with the approximately equitoxic VRL (IC15) concentration (slight synergism). However, in CAL-27 cells, IC45 concentrations of VRL already caused a more pronounced radiosensitising effect (DEF = 1.92, synergistic effect) compared to the equitoxic VFL concentration (DEF = 1.41, only an additive effect). MCF-7 was the only cell line in which the radiosensitising effect of VRL was not concentration-dependent, in contrast to the results obtained with VFL.

Regarding the cell cycle effects of VRL, a statistically significant concentration-dependent G2/M block was observed after 24 h incubation (timepoint of radiation in the chemoradiation experiments). A significant G2/M block was already seen after short incubation times (4 h), a maximum was reached after 16 h, followed by a significant decrease in the amount of G2/M phase cells, resulting in recycling of the cells in a normal or polyploid cell cycle. After removal of VRL, cells also started recycling very rapidly (from 3 h after removal). After 24 h incubation and especially after a prolonged continuous incubation (48 h), a polyploid population was clearly observed in ECV304 and CAL-27 cells (p-value < 0.05 compared to control), and less pronounced in the other cell lines. A similar progress through the cell cycle was observed with VFL treatment, with small (sometimes statistically significant) cell line-dependent differences [30].

Our results obtained with VRL, confirmed and extended the results already described in the literature. Four human tumour cell lines, among which also a bladder and a breast cancer cell line next to a lung and head & neck cancer cell line, were tested simultaneously. Edelstein et al. investigated 2 human lung carcinoma cell lines (NCI-H460 & A549) and concluded that 24 h incubation with VRL before or after radiation resulted in a dose-dependent potentiation of radiation, which was also cell cycle-dependent, with maximal effect when cells were in the G2 phase of the cell cycle [26]. Also PC9 NSCLC cells were sensitised to radiation by VRL, by causing accumulation of cells in the G2/M phase of the cell cycle. Prolonged G2/M accumulation concomitant with continuous polyploidisation and increased susceptibility to induction of apoptosis may be associated with the cellular mechanism of radiosensitisation produced by VRL in this cell line [27]. As described by Fukuoka et al., SBC-3 cells, human SCLC cells, were also sensitised to radiation by VRL and a possible mechanism of the VRL-induced radiosensitisation may in part, be associated with impairment of DNA repair following radiation-induced DNA damage. It was hypothesized that the disruption of microtubule integrity in SBC-3 cells by VRL in part might inhibit p53 transport to the nucleus, resulting in impairment of p53-mediated DNA repair following radiation-induced DNA damage [28]. Erjala et al. investigated the concomitant use of vinorelbine and radiation in 8 head and neck squamous cell carcinomas (HNSCC). An additive effect (but not supra-additivity) with radiation was noticed in all cell lines tested [29]. The results described in the literature to date, are cell line-dependent and several mechanisms for the radiosensitising effect are proposed.

In view of our results, the observed G2/M block can be related to the radiosensitising effect of VRL after 24 h incubation followed by radiation. Also the contribution of the polyploid population may be important. In ECV304 and CAL-27, a statistically significant increase in S_2 _and (G2/M)_2 _was already observed after 24 h incubation, although in H292 and MCF-7 this was only the case after a prolonged incubation (48 h). Further investigation of the polyploid population seems appropriate and of interest. Possible contributions of DNA repair mechanisms or the susceptibility to apoptosis warrants further study.

Overall, our study indicates that the radiosensitising effects of VFL and VRL *in vitro *are comparable (p-value comparing VRL with VFL never below 0.01), with small, statistically insignificant cell line-related differences, and indeed the cell cycle effects (G2/M block, polyploidisation) can be related to the observed radiosensitising effects. Preclinical studies with VRL have prompted further study of VRL as a radiosensitiser in the clinic. A Phase I study of radiotherapy to the thorax combined with daily VRL (4–6 mg/m^2^) administration (as a radiosensitiser) in 14 patients with locally advanced NSCLC resulted in 4 partial responses and 2 complete responses [38]. Also the combined use of VRL with platinum compounds and irradiation has been shown to be feasible. The survival rates achieved with this approach appeared to be superior to those achieved with radiotherapy alone [39]. VFL has not been studied in this setting. However, the advantages of VFL over VRL are the following: (1) it has definite superior antitumour activity against a wide range of experimental tumour models compared to VRL, (2) it proved to be a far less potent inducer of resistance than VRL, (3) it shows a high level of synergy when combined with other chemotherapeutic agents, (4) it has anti-vascular and anti-angiogenic effects at doses below the MTD, (5) it is extremely well tolerated by patients used in a weekly schedule [40]. Combined with a reduced neurotoxicity related to VRL (Table 6), these characteristics make VFL an interesting addition to the currently available armamentarium of chemotherapeutic agents, and, potentially an interesting candidate for chemoradiation studies.

**Table 6:** Comparison between vinflunine and vinorelbine regarding their preclinical effects and their clinical implications.

PRECLINICAL STUDIES:	
	
*IN VITRO*	VFL vs VRL
- mechanism of action^3,17^	equal
- radiosensitising effect	equal
- cell cycle effect	equal
- cross-resistance to other MDR-inducing drugs^41^	VFL: least cross-resistant
- inducer of drug resistance^15,20^	VFL far less potent than VRL at 2 × IC50: resistance after 8 months instead of within 2 weeks for VRL
- combination with other chemotherapeutic agents^42^	VFL: high level of synergy
	
*IN VIVO*	
- efficacy against a series of murine and human tumour experimental models^15,16^	VFL definite superiority to VRL
	VFL: high or moderate activity in 64% (7 of 11)
	VRL: moderate activity in 27% (3 of 11)
- inducer of drug resistance^20^	VFL far less readily than VRL
	10 mg/kg vs 2.5 mg/kg – P388: complete resistance after 22 weeks instead of after 5 weeks
- tolerance^15,43,44^	VFL: high level, superior to VRL
- anti-vascular effects^44,45^	VFL: at doses much lower (5-fold) than the MTD
- anti-angiogenic effects^45^	VFL: at doses below the optimum effective single dose (40-20-fold lower than its MTD)
- activity against metastases^45^	VFL: significant effects at low doses (16-fold lower than the MTD)
	
CLINICAL IMPLICATIONS:	
- neurotoxicity^5,6,17,18,46^	VFL: reduced relative to VRL
- therapeutic window^6,16,45,47^	VFL: presumed to be wider than VRL

## Conclusion

The radiosensitising effects of VFL and VRL were not statistically different from each other and were nearly always cell line-specific and concentration-dependent. The cell cycle effects could be related to the observed radiosensitising effects. Considering the more favourable toxicity profile of VFL, this agent might be more promising than VRL for chemoradiation studies in the clinic.

## Competing interests

F. Breillout is employed by 'Institut de Recherche Pierre Fabre'. All other authors declare that they have no competing interests.

## Authors' contributions

CS participated in the design of the study, performed all the experiments and drafted the manuscript. FL, BP and JBV participated in the conception, design and coordination of the study, and revised the manuscript critically. CDP was involved in the irradiation experiments. HL and GP participated in the cell survival experiments and performed cell culture. FB contributed to the conception of the study and revised the manuscript critically. All authors have read and approved the final manuscript.

## Pre-publication history

The pre-publication history for this paper can be accessed here:


